# Epidemiology investigation and analysis of patients with hemodialysis in Sichuan province of China

**DOI:** 10.1080/0886022X.2019.1612429

**Published:** 2019-07-11

**Authors:** Yang Zou, Daqing Hong, Qiang He, Yu Wen, Guisen Li

**Affiliations:** a Department of Nephrology, Sichuan Academy of Medical Sciences & Sichuan Provincial People’s Hospital, Chengdu, China;; b Department of Nephrology, Sichuan Provincial Ziyang People’s Hospital, Ziyang, China

**Keywords:** Epidemiology, end-stage renal disease, hemodialysis, Chinese National Renal Data System, Sichuan province

## Abstract

**Objectives:** To investigate the incidence, pathogenesis, as well as mortality rate and causes of end-stage renal disease patients with hemodialysis (ESRD-HD) in Sichuan province of China.

**Methods:** In this retrospective descriptive study, all the data were exported from the Chinese National Renal Data System (CNRDS). The demographic and pathogenic information from 01 January 2011 to 31 December 2016 were statistically analyzed.

**Results:** According to the data from CNRDS, the incidence of ESRD-HD was high in Sichuan province. From 2011 to 2016, the annual incidence rate of ESRD-HD was 61.84, 73.75, 78.04, 66.04, 72.61, and 60.98 per million population (pmp). Male ESRD-HD patients were higher than female patients (1.5:1). The major causes of ESRD-HD in Sichuan province from 2011 to 2016 were chronic glomerulonephritis and diabetic nephropathy. The annual mortality rate of ESRD-HD was 113.20, 91.25, 73.02, 68.56, 68.57, and 58.39 per 1000 person-years. The descending rate of mortality was parallel in both males and females. The mortality rate was observed to be higher in the elderly group (age ≥ 60). The major cause of mortality was cardiovascular diseases (24.48%).

**Conclusions:** In Sichuan province, the incidence of ESRD-HD annually was gradually descending. The elderly and male patients had higher incidence of ESRD-HD. The annual mortality rate of ESRD-HD was declining year by year, and elderly patients aged ≥ 60 contributed to the highest mortality rates. The major cause of death was cardiovascular diseases. This could contribute to a better understanding of ESRD-HD in southwest of China, thus providing better treatment for ESRD in the future.

## Introduction

Increasing incidence of end-stage renal disease (ESRD) and its associated morbidity is a serious worldwide public health problem. According to the 2017 US Renal Data System (USRDS) Annual Data Report, the number of new ESRD cases in the United States was 124 111 in 2015 [1]. So, with the increasing ESRD cases, the demand for renal replacement therapy (RRT) is increasing year by year. The choice of predominant method of RRT varies in different countries. In China, about 86% of the dialysis population receive hemodialysis (HD) [2], and about 72.8%, 84% of the dialysis population receive HD in Zhejiang province, Shanghai of China, respectively [2,3]. HD is still considered as a major treatment choice for patients in Sichuan province in southwest of China, and receive HD twice or thrice weekly.

The number of patients requiring dialysis is rapidly increasing in the past 10 years, but HD is an expensive treatment modality that lays a heavy burden on the national healthcare cost. In China, the ERSD patients are increasing by 120 thousand each year, which requires more than 1.4 billion dollars for the maintenance of their survival and treatment. This accounted for 1–3% of national healthcare costs and presented consecutive stress on the nation’s health foundation [4].

Hence, it is imperative to understand the incidence, pathogenesis and mortality rate in end-stage renal disease patients with hemodialysis (ESRD-HD) patients according to gender and age from 2011 to 2016 in Sichuan province. Our results are considered important for the government to deal with the increasing burden of patients with ESRD-HD.

## Clinical materials and methodology

### Data sources and ethnics

The Chinese National Renal Data System (CNRDS) is initiated by the Chinese Society of Nephrology under the lead of the Ministry of Health of China in 2010. The main objectives of the CNRDS include collection of demographic, clinical and laboratory data of HD and peritoneal dialysis (PD) cases to establish a kidney disease database for research and policy making [2]. The data for retrospective analysis were exported from the CNRDS, including new, long-term and dead ESRD-HD patients from 01 January 2011 to 31 December 2016 in south west of China, Sichuan province. This study was approved by the Hospital Human Research Ethics Committee of Sichuan Academy of Medical Sciences & Sichuan Provincial People’s Hospital (No. 2017-120).

### Analyzed indexes

This was a retrospective descriptive study. All the demographic and pathogenic information of the patients with ESRD-HD were extracted from the CNRDS, which included the demographics of ESRD-HD patient’s, primary causes as well as the death rates of ESRD-HD patient’s, along with the causes of death. Analysis included annual morbidity, gender differences, and pathogenesis, as well as mortality rates and causes. The comparison and differences between different sexes and ages were emphatically analyzed by statistical methods.

The annual incidence was calculated as the number of new patients undergoing HD between 1 January and 31 December divided by the provincial resident population. The obtained value was expressed as per million population (pmp).

The mortality rates (per 1000 person years) were calculated as the number of HD deaths between 1 January and 31 December divided by mid-year prevalent dialysis patients (the number of prevalent dialysis patients at the end of year (*x* – 1)) + (the number of prevalent dialysis patients at the end of year *x*)/2.

### Statistical methodology

Statistical analysis of the data was performed by using SAS (version 9.3, Cary, NC). The measurement data were expressed as mean ± standard deviation. Enumeration data were expressed as case numbers (percent). Comparison between the two groups was done by using Chi square test or Fisher’s exact test. *p* Values of <.05 were considered to be statistically significant.

## Results

### Patient characteristics of incident ESRD-HD

According to the data from CNRDS, there were 33 632 patients with an incidence of ESRD-HD from 01 January 2011 to 31 December 2016. The incidence of ESRD-HD annually was 61.84, 73.75, 78.04, 66.04, 72.61, and 60.98 pmp each year, respectively (Table 1).

**Table 1.** The incidence of initial ESRD-HD patients from 2011 to 2016 in Sichuan province (pmp).

Year	2011	2012	2013	2014	2015	2016
Initial ESRD-HD patients	4978	5956	6327	5376	5957	5038
Resident population (million)	80.50	80.76	81.07	81.40	82.04	82.62
Incidence of ESRD-HD (pmp)	61.84	73.75	78.04	66.04	72.61	60.98
Initial ESRD-HD patients (male)	2989	3603	3758	3140	3526	2981
Resident population (million, male)	41.12	42.3	41.33	40.34	41.20	41.37
Incidence of ESRD-HD (pmp, male)	72.69	85.18	90.93	77.84	85.58	72.06
Initial ESRD-HD patients (female)	1989	2353	2569	2236	2431	2057
Resident population (million, female)	39.38	38.46	39.74	41.06	40.84	41.25
Incidence of ESRD-HD (pmp, female)	50.51	61.18	64.65	54.46	59.53	49.87

Of the total incident HD patients, males accounted for 19 997 and females accounted for 13 635, with a ratio of 1.5:1 (male:female). The incidence of ESRD-HD patients undergoing HD was higher in males than in females (*p* < .05, Table 1).

There were 32 952 HD patients with complete information of age, accounting for 98.0% of the total. It was also noted that the middle age and elder group contributed to most of the incidence of HD patients (Table 2).

**Table 2.** The incidence of ESRD-HD in patients by different ages from 2011 to 2016 in Sichuan province (%).

Age/year	2011	2012	2013	2014	2015	2016	2011–2016
<18	28 (0.6%)	31 (0.5%)	55 (0.9%)	23 (0.4%)	18 (0.3%)	23 (0.5%)	178
18–29	429 (9.1%)	565 (9.7%)	531 (8.4%)	398 (7.5%)	430 (7.3%)	303 (6.2%)	2656
30–39	749 (15.9%)	845 (14.4%)	775 (12.3%)	545 (10.3%)	535 (9.1%)	431 (8.7%)	3880
40–49	1116 (23.7%)	1446 (24.7%)	1533 (24.4%)	1292 (24.3%)	1336 (22.8%)	1023 (20.8%)	7746
50–59	764 (16.2%)	938 (16.0%)	1047 (16.7%)	995 (18.7%)	1131 (19.3%)	1049 (21.3%)	5924
60–69	892 (19.0%)	1092 (18.7%)	1297 (20.6%)	1142 (21.5%)	1281 (21.8%)	1108 (22.5%)	6812
70–79	587 (12.5%)	741 (12.7%)	818 (13.0%)	725 (13.7%)	889 (15.2%)	790 (16.0%)	4550
≥80	140 (3.0%)	193 (3.3%)	233 (3.7%)	192 (3.6%)	245 (4.2%)	203 (4.1%)	1206
Total	4705	5851	6289	5312	5865	4930	32 952

### Pathogenesis analysis of incident ESRD-HD patients

**Figure 1.**
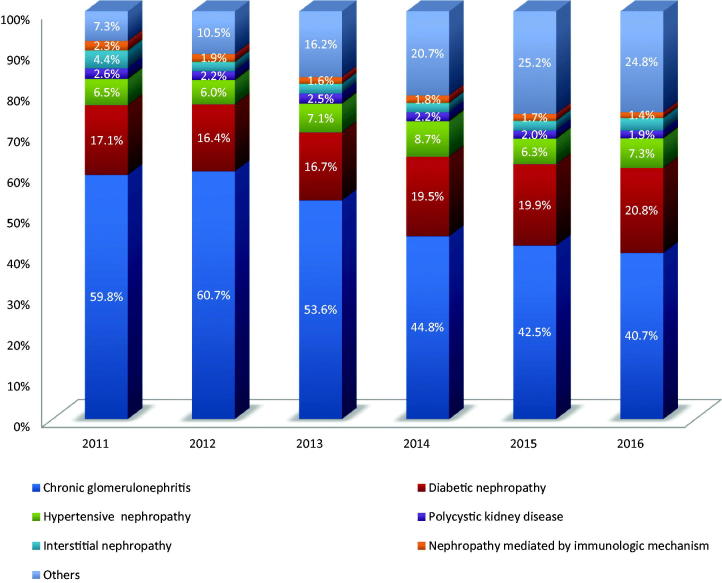
The pathogenesis analysis incidence of ESRD-HD patients from 2011 to 2016 in Sichuan province (%).

Among the 33 632 incident ESRD-HD patients, there were 29 447 cases with complete information of pathogenesis, accounting for 87.7% of the total. The major causes of incident ESRD-HD included chronic glomerulonephritis, diabetic nephropathy, and hypertensive nephrosclerosis. The percentage of chronic glomerulonephritis was gradually descending annually, whereas the percentage of diabetic nephropathy was ascending (Figure 1).

*Other*: Obstructive nephropathy, drug-induced renal injury, hereditary kidney disease, hyperuricemia, and so on.

### Mortality analysis of ESRD-HD patients

According to the data from CNRDS, there were 8285 deaths from 01 January 2011 to 31 December 2016 in Sichuan province of China. The annual mortality rate of ESRD-HD patients was 113.20, 91.25, 73.02, 68.56, 68.57, and 58.39 per 1000 person-years from 2011 to 2016. The descending rate of mortality was parallel in both males and females (Table 3).

**Table 3.** The mortality of ESRD-HD patients and from 2011 to 2016 in Sichuan province (per 1000 person-years).

Year	2011	2012	2013	2014	2015	2016
ESRD-HD patients	10 248	13 425	17 310	20 830	23 948	26 820
Death number of ESRD-HD patients	1160	1225	1264	1428	1642	1566
Mortality rate of ESRD-HD (per 1000 person-years)	113.20	91.25	73.02	68.56	68.57	58.39
ESRD-HD patients (male)	6070	8033	10 307	12 378	14 232	15 940
Death number of ESRD-HD patients (male)	675	722	766	820	931	936
Mortality rate of ESRD-HD (male.per 1000 person-years)	111.2	89.89	74.32	66.25	65.42	58.72
ESRD-HD patients (female)	4178	5441	7003	8452	9716	10 880
Death number of ESRD-HD patients (female)	485	503	498	608	711	630
Mortality rate of ESRD-HD (female.per 1000 person-years)	116.08	92.45	71.11	71.94	73.18	57.9

Among all the dead patients (8285), 7828 cases (80.0%) completely registered their age. Most of the deaths were encountered in the elderly group (age ≥ 60) (Table 4).

**Table 4.** The constitution of dead ESRD-HD patients by different ages from 2011 to 2016 in Sichuan province (%).

Age/year	2011	2012	2013	2014	2015	2016	2011–2016
<18	2 (0.2%)	1 (0.1%)	2 (0.2%)	3 (0.2%)	3 (0.2%)	2 (0.1%)	13
18–29	40 (4.1%)	44 (3.8%)	30 (2.5%)	36 (2.6%)	31 (2.0%)	28 (1.9%)	209
30–39	91 (9.2%)	84 (7.3%)	71 (5.8%)	53 (3.8%)	68 (4.3%)	43 (2.9%)	410
40–49	174 (17.7%)	183 (15.9%)	195 (15.9%)	211 (15.2%)	210 (13.3%)	200 (13.3%)	1173
50–59	148 (15.0%)	159 (13.9%)	196 (16.0%)	189 (13.6%)	281 (17.8%)	233 (15.5%)	1206
60–69	235 (23.8%)	290 (25.3%)	323 (26.4%)	353 (25.4%)	376 (23.9%)	422 (28.1%)	1999
70–79	230 (23.3%)	277 (24.2%)	301 (24.6%)	388 (27.9%)	432 (27.4%)	403 (26.8%)	2031
≥80	66 (6.7%)	109 (9.5%)	108 (8.8%)	156 (11.2%)	175 (11.1%)	173 (11.5%)	787
Total	986	1147	1226	1389	1576	1504	7828

**Figure 2.**
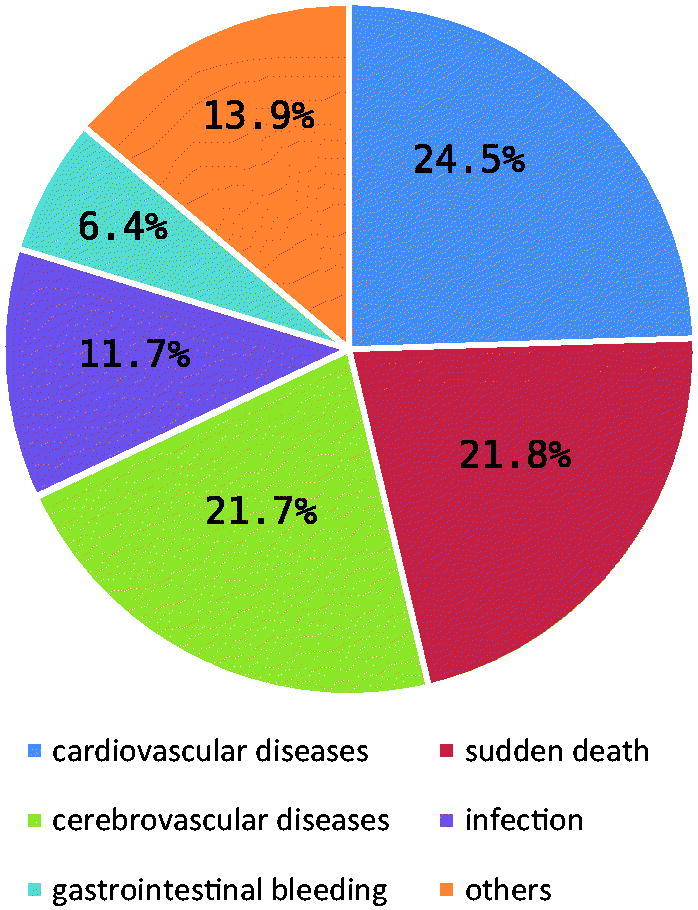
The constitution of causes of death for ESRD-HD patients from 2011 to 2016 (%).

Among all the causes of death (8285), 6630 cases (80.0%) were completely registered. The major causes of death were cardiovascular diseases (24.5%, 1623/6630), sudden death (21.8%), cerebrovascular diseases (21.7%), infections (11.7%), and gastrointestinal bleeding (6.4%) (Figure 2). Heart-failure accounted for 34.4% (558 of 1623) in cardiovascular diseases, and cerebral hemorrhage accounted for 75.1% (1082 of 1440) in cerebrovascular diseases.

## Discussion

ESRD affects a large population, leading to a high prevalence worldwide [5,6]. Zhang et al. showed that the prevalence of chronic kidney disease (CKD) was 10.8% in China, but its prevalence was 18.3% in Sichuan province [7]. According to other reports, 2% CKD patients have progressed to ESRD [8]. Current treatment for ESRD includes HD, PD, and kidney transplantation [9,10]. In most of the countries and regions, HD is the major treatment for ESRD patients. In China, HD is still considered the major strategy for ESRD treatment. Dialysis patients have lower survival rates when compared to the general population [11–13]. The mortality rate of HD patients is 6.1–7.8 folds in the general population of same age, and the USRDS data showed that the survival rate of 5 year-dialysis patients is as low as 49% [14]. So, ESRD has become a significant public health issue worldwide.

Our study demonstrated the trends of increasing ESRD incidence in Sichuan population. The results showed that the annual incidence of new HD patients from 2011 to 2016 was 61.84, 73.75, 78.04, 66.04, 72.61, and 60.98 pmp, respectively. Generally, the annual incidence of new HD patients in Sichuan is lower than the well-developed areas of China such as Beijing (99.9–153.4 pmp) [15], Shanghai (114.8 pmp) [16], and higher than the developing areas such as Shanxi province (46.62–62.93 pmp) [17] and Hainan province (44.2–64.7 pmp) [18].

The possible reasons for these differences could be due to different financial and health care levels. Also, there could be loss of some information during the registration process of the patients, leading to underestimation of the annual incidence.

We found that the incidence of HD patients with ESRD-HD in Sichuan province was different between males and females. The sex ratio was 1.5:1 (male:female). This was consistent with other reports, where there were 1.3 million females and 1.7 million males who were undergoing treatment with dialysis. It should be noted that the women’s access to medical care in many countries was substantially limited due to lack of universal health coverage, high health care expenses, low income [19], as well as women’s social roles as non-wage earners in some patriarchal societies. Biological factors responsible for higher male prevalence rates for dialysis included the damaging effects of testosterone in men [20]. Due to the protective effects of estrogen in women, the incidence rate of ESRD-HD is lower compared to men [20].

Our study demonstrated that the elderly group included more number of new HD patients. This might be due to the aging of population in both developed as well as undeveloped countries and areas, and have chosen the option of HD for treatment. Indeed, we noticed that chronic glomerulonephritis, diabetic nephropathy, and hypertensive nephrosclerosis were the major causes of ESRD-HD in Sichuan province. Unlike the well developed countries, glomerulonephritis is the first cause of CKD in China. Among the patients who are undergoing dialysis, glomerulonephritis accounted for the highest cause, followed by diabetic nephropathy [21]. Barsoum reported that the high prevalence of bacterial and viral infection affect the kidney; therefore, chronic glomerulonephritis currently is the principal cause of CKD in developing countries such as China, India, and Egypt [22]. It is worth noting that the histologic types of glomerulonephritis in the developing countries vary considerably. IgA nephropathy predominates in China and it accounts for 37–58% of biopsy-proven primary glomerulonephritis [23]. However, we found that the percentage of patients with chronic glomerulonephritis is descending and that of diabetic nephropathy is rising. With the changing way of life in China, the incidence of diabetes is also rising, and currently China is ranked in the first place in the world. Unlike the western countries, most of the diabetic patients in China are young and middle-aged, and these patients are exposed to high doses of blood glucose for a longer period, causing more damage to the kidney [21].

Our study showed that the annual mortality rate of ESRD-HD was 113.20, 91.25, 73.02, 68.56, 68.57, and 58.39 per 1000 person-years from 2011 to 2016, which showed a descending trend. This was consistent with the USRDS data from the US [1].

Moreover, our results showed that death caused by cardiovascular diseases was declining. As known, cardiovascular diseases are the leading causes of mortality in ESRD-HD patients. So, individuals should pay more and more attention to the cardiovascular diseases. As the number of deaths induced by cardiovascular diseases decrease, the mortality rates of HD patients reduce [24].

Finally, our study showed that the elderly HD patients accounted for 61.5% of the total death population. Dialysis outcomes and practice patters (DOPP) reported that the HD patients of >65 years have a mortality risk of 100% higher than the younger population [25]. So, more attention should be paid to this age group.

In conclusion, the epidemiological manifestations and pathogenesis analysis of HD patients with ESRD-HD, mortality rates and causes of ESRD-HD in southwest of China, Sichuan province were presented. These data suggested for the urgent development and implementation of effective ESRD prevention strategies, especially in the elderly and diabetic nephropathy patients.
